# Apelin facilitates integrin αvβ3 production and enhances metastasis in prostate cancer by activating STAT3 and inhibiting miR-8070

**DOI:** 10.7150/ijbs.113161

**Published:** 2025-06-12

**Authors:** Tien-Huang Lin, Shan-Chi Liu, Yuan-Li Huang, Chao-Yang Lai, Xiu-Yuan He, Chun-Hao Tsai, Yi-Chin Fong, Hsi-Chin Wu, An-Chen Chang, Yen-You Lin, Chih-Hsueh Lin, Chih-Hsin Tang

**Affiliations:** 1 School of Post-Baccalaureate Chinese Medicine, Tzu Chi University, Hualien, Taiwan.; 2 Department of Urology, Buddhist Tzu Chi General Hospital Taichung Branch, Taichung, Taiwan.; 3 Institute of Biomedical Sciences, Mackay Medical College, New Taipei City, Taiwan.; 4 Department of Medical Laboratory Science and Biotechnology, Asia University, Taichung, Taiwan.; 5 Department of Pharmacology, School of Medicine, China Medical University, Taichung, Taiwan.; 6 Department of Sports Medicine, College of Health Care, China Medical University, Taichung, Taiwan.; 7 Department of Orthopedic Surgery, China Medical University Hospital, Taichung, Taiwan.; 8 Department of Orthopedic Surgery, China Medical University Beigang Hospital, Yunlin, Taiwan.; 9 School of Medicine, China Medical University, Taichung, Taiwan.; 10 Department of Urology, China Medical University Hospital, Taichung, Taiwan.; 11 Department of Urology, China Medical University Beigang Hospital, Beigang, Yunlin, Taiwan.; 12 School of Oral Hygiene, College of Oral Medicine, Taipei Medical University, Taipei, Taiwan.; 13 Translational Medicine Center, Shin Kong Wu Ho-Su Memorial Hospital, Taipei, Taiwan.; 14 Chinese Medicine Research Center, China Medical University, Taichung, Taiwan.; 15 Department of Family Medicine, China Medical University Hospital, Taichung, Taiwan.

**Keywords:** prostate cancer, apelin, metastasis, integrin, miR-8070

## Abstract

Prostate cancer is the most common cancer in men and is often associated with distant metastasis in its later stages. Cell surface receptors called integrins function as adhesion molecules that mediate both cell adhesion and motility. The adipokine apelin has been implicated in cancer progression and metastasis. However, the mechanisms by which apelin regulates integrin production and metastasis in prostate cancer remain unclear. Here, we found that apelin and integrin αvβ3 expression levels were elevated in prostate cancer samples compared to those in normal individuals. Apelin stimulation enhances integrin αvβ3-dependent prostate cancer migration. The activation of STAT3 and inhibition of miR-8070 via the MAPK pathway mediate apelin-facilitated integrin synthesis and cell motility. Importantly, our *in vivo* study indicated that inhibiting apelin reduces integrin αvβ3 expression and prostate cancer metastasis. Our results suggest that the apelin/integrin axis is a novel therapeutic target for treating metastatic prostate cancer.

## Introduction

The most prevalent disease in males is prostate cancer, whose risk rises with age and fat 1, 2. It has been predicted that by 2050, there would be more than 300,000 new cases detected annually in the United States of America alone. More than half of patients with prostate cancer eventually develop bone metastases, especially in advanced stages, although many people initially present with localized disease 2, 3. Many patients with metastatic prostate cancer experience treatment resistance, even though the five-year relative survival rate for all diagnosed instances of the illness is 98%. These metastases are a major cause of death and can drastically lower a patient's quality of life 4. A thorough understanding of the intricate processes behind the spread of prostate cancer and distant metastases is essential for future advancements in early prevention and intervention.

The process by which tumor cells leave their original site, move through the bloodstream, and develop into a new tumor in a different organ is known as metastasis. Cell surface receptors called integrins function as adhesion molecules that can mediate connections between the extracellular matrix and prostate epithelial cells 5. Integrin heterodimers made up of α and β subunits connected by noncovalent bonds are seen in normal prostate basal cells 6. There are a total of 24 distinct pairings formed by the 18 α and 8 β subunits that have been found thus far 7, 8. Cell adhesion and motility are modulated by integrins in a range of biological processes 7. The progression, migration, invasion, and metastasis of prostate cancer cells are linked to the integrins α2β1, αvβ3, α5β1, and α6β1 9, 10.

The *APLN* gene encodes the peptide known as apelin 11, which is released by adipose tissue and contains a 77-amino acid preprotein precursor 12. Numerous biological processes, such as angiogenesis 13, blood pressure regulation 14, and cellular metabolism 15, have been linked to the different molecular forms of apelin. Additionally, apelin has been linked to the pathological advancement of cancer, obesity, and diabetes 16. By controlling variables related to tumor growth and motility, apelin influences tumor development and metastasis 17. It has been demonstrated that apelin causes tumor growth, migration, and invasion in ovarian and gastric cancer, cholangiocarcinoma, lung cancer, and oral squamous cell carcinoma 18. We previously demonstrated that apelin enhances prostate cancer motility 19; however, the effects of apelin on integrin expression and distant metastasis of prostate cancer remain unclear. Here, we found that apelin and integrin αvβ3 are upregulated in prostate cancer patients compared to healthy individuals. Apelin enhances wound healing and cell migration in prostate cancer by promoting integrin αvβ3 production. Activation of STAT3 and inhibition of miR-8070 through the MAPK pathway are involved in apelin-induced prostate cancer migration. Inhibiting apelin reduces prostate metastasis *in vivo*, indicating that the apelin/integrin αvβ3 axis is a promising target for treating prostate cancer metastasis.

## Materials and Methods

### Materials

JNK (SC-474), p38 (SC-4972), ERK (SC-1647), STAT3 (SC-482), p-JNK (SC-6254), p-p38 (SC-166182), p-ERK (SC-7383) and integrin αvβ3 (SC-7312) antibodies as well as JNK (sc-29380), p38 (sc-29433), STAT3 (sc-29493), integrin αv (sc-29373), integrin β3 (sc-29375) and apelin (sc-44741) siRNA were purchased from Santa Cruz Biotechnology (CA, USA). Antibody against the phosphorylated form of STAT3 (AP0705) was obtained from Abclonal technology (Woburn, MA, USA). Apelin antibody was acquired from NOVUS biologicals (Centennial, CO, USA). Recombinant human apelin was acquired from PerpoTech (Rocky Hill, NJ, USA). All other chemicals utilized in this study were obtained from Sigma-Aldrich (St. Louis, MO, USA).

### Cell culture

The American Type Culture Collection provided the human prostate cancer cell lines (PC3 and DU145). Both of PC3 and DU145 cells are Androgen-insensitive cell lines, while PC3 cells showed higher metastatic potential compared to DU145 cells. In RPMI-1640 media supplemented with 20 mM HEPES, 10% heat-inactivated fetal calf serum, 2 mM glutamine, 100 U/ml penicillin, and 100 μg/ml streptomycin, cells were kept at 37 °C with 5% CO_2_.

### Bioinformatics analysis

Apelin levels were analyzed using the GSE6919 dataset obtained from the Gene Expression Omnibus (GEO) database 20. This dataset provided expression levels of target genes in healthy prostate tissue and prostate tumor tissues. The GSE7930 dataset compared integrin αv and β3 levels between highly metastatic and poorly metastatic tumors from prostate cancer patients. The GSE7930 dataset was formatted and uploaded to QIAGEN Ingenuity Pathway Analysis (IPA) software (Hilden, Germany) for further exploration. The imported datasets were analyzed using the “Core Analysis” module and filtered for “gene expression.” Analysis of mRNA expression changes in IPA identified pathways with a strong positive correlation to prostate cancer. miRNA candidates were mined from the miRWalk database (Hilden, Germany). To identify miRNA regulators, the official gene symbols for integrin αv and β3 were used in the search process. To accurately identify miRNAs binding to integrin αv and β3, results were filtered using TargetScan and miRDB. After the data processing phase, subsequent experiments enabled us to pinpoint specific miRNAs regulated by apelin.

### Real-time quantitative PCR analysis

The TRIzol reagent (MDBio; Taipei, Taiwan) was used to extract total RNA from prostate cancer cells. In summary, oligo-dT primers were used to reverse-transcribe 1 μg of RNA into cDNA in compliance with the manufacturer's instructions (Invitrogen; Carlsbad, CA, USA). The Mir-XTM miRNA First Strand Synthesis Kit (Takara Bio Inc.) was used to create cDNA from 100 ng of total RNA for the miRNA assay. The endogenous control gene was used to achieve relative quantification of gene expression. The fractional cycle number at which the fluorescence exceeded the predetermined threshold was known as the threshold cycle (CT). The comparative CT approach was used to calculate relative expression 21, 22. The primer sequence was provided in Supplementary Table S1.

### Western blot analysis

After electrophoretic separation on SDS-PAGE gels (7.5-12%), protein samples were transferred onto PVDF membranes (Merck; Darmstadt, Germany). Before the membranes were incubated with primary antibodies for an entire night at 4°C, they were blocked using 5% nonfat milk. After that, the membranes were treated for an hour at room temperature with certain secondary antibodies. An ECL kit (Millipore, USA) was used to detect the target protein's expression, and an ImageQuantTM LAS 4000 biomolecular imager was used to visualize it 23, 24.

### Cell transfection

After seeding the cells (5 × 10⁵ cells/well) onto 6-well plates, the cells were transfected with different miRNA mimics or genetic siRNAs using Lipofectamine 2000 transfection reagent (Waltham, Massachusetts, USA) in accordance with the manufacturer's instructions.

### Prostate cancer migration assay

Transwell inserts (Costar, NY; 8-mm pore size) were used in 24-well plates for the migration experiment. Cells were pretreated with varying inhibitors for 30 minutes before to the migration assay. The upper compartment contained about 1 × 10^4^ cells in 200 μl of serum-free media, whereas the lower chamber had 300 μl of the same medium with varying apelin concentrations. The cells were fixed in 3.7% formaldehyde for five minutes, incubated for twenty-four hours at 37 °C in 5% CO_2_, and stained with 0.05% crystal violet in PBS. Cotton-tipped swabs were used to remove cells from the upper side of the filters, and PBS was used to wash the filters 25.

### Wound-healing migration assay

Cells were seeded at a density of 1 × 10^5^ cells/well in culture media onto 12-well plates for the wound-healing migration test. The monolayer cells were manually scratched with a pipette blue tip 24 hours after seeding in order to make long, distinct scratches in the middle of the dishes with a clear, brilliant field. The cells were washed once with PBS in order to eliminate the unattached cells. Each dish was supplemented with serum-free media, either with or without apelin. The photos were obtained for each point after a 24-hour migration, and the number of migratory cells was then counted and averaged for each experimental condition.

### Immunohistochemistry (IHC)

Immunohistochemistry assays were conducted on tissue specimens obtained from prostate cancer patients and mouse tissues. The primary antibodies employed in the immunohistochemistry procedure were against apelin and integrin αvβ3. Apelin and integrin αvβ3 expression illustrated on the tumor of prostate cancer patients or mouse tissue were quantified by Fiji software. The darker 3, 3'-diaminobenzidine (DAB) expression gained the higher OD value during quantified procedure. Then, the sum of the OD values, which were quantified from the pathological section, divided by the area of ​​the target distribution area to obtain the average optical density (AOD) value. The AOD value reflects the concentration per unit area of ​​the target genes 26.

### Statistics

All values are presented as mean ± standard deviation (SD). Statistical significance between experiment groups was assessed using the student's t-test. Comparisons involving more than two groups with a single variable were performed using one-way analysis of variance (ANOVA) followed by Bonferroni's *post hoc* test. Differences between groups were considered significant if the *p*-value was < 0.05.

## Results

### Apelin promotes prostate cancer migration through upregulating integrins αvβ3

**Figure 1:**
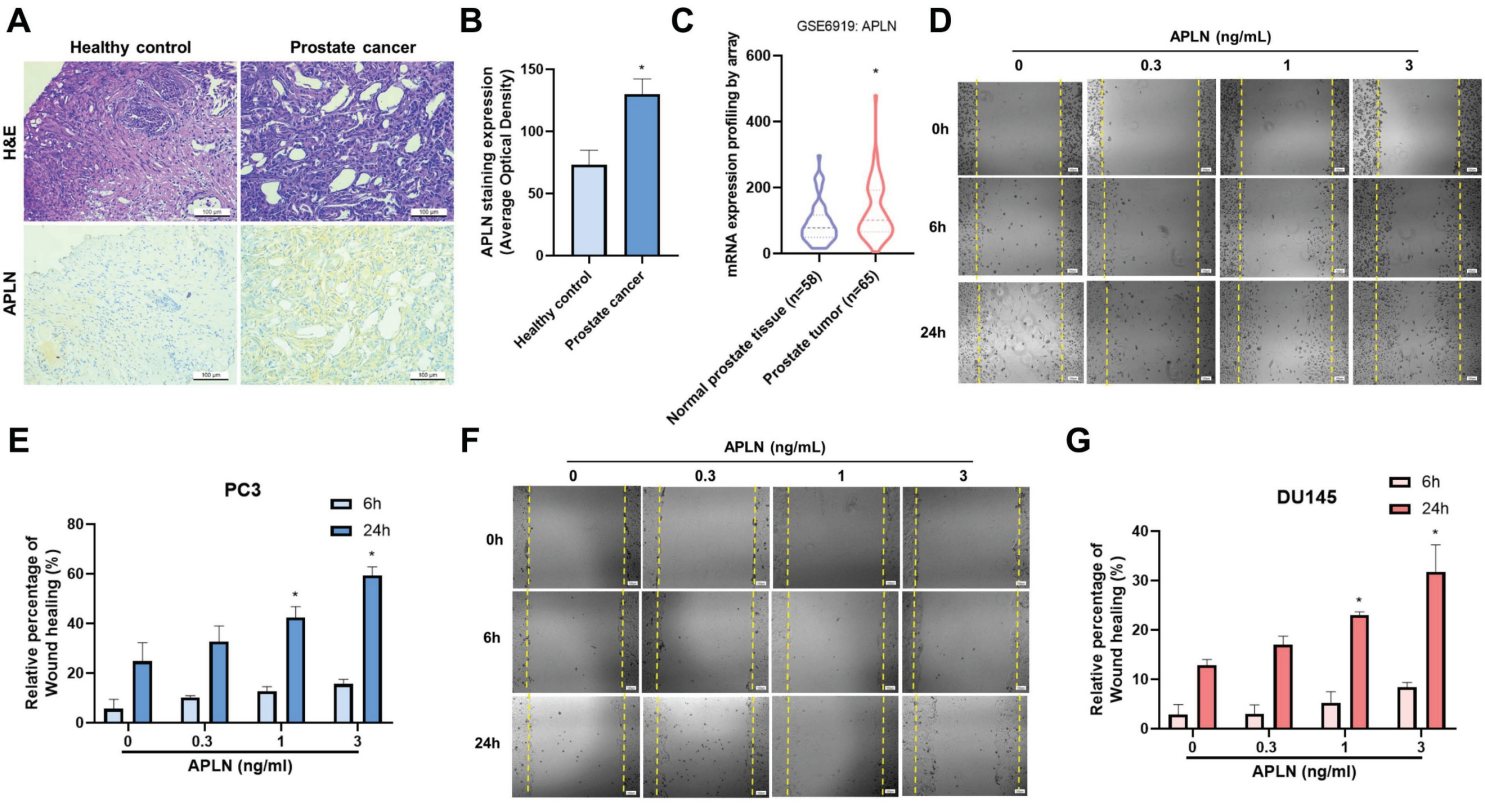
** Apelin promotes wound healing migration in prostate cancer cells.** (A&B) Representative images showing the results of IHC staining for apelin in tissue samples from healthy individuals (n=3) and prostate cancer patients (n=3). (C) Apelin gene levels in normal and prostate cancer patients retrieved from the GEO database. (D-G) Cells were treated with apelin, the wound healing migration was examined (n=3). * *p* < 0.05 compared with the control group.

**Figure 2:**
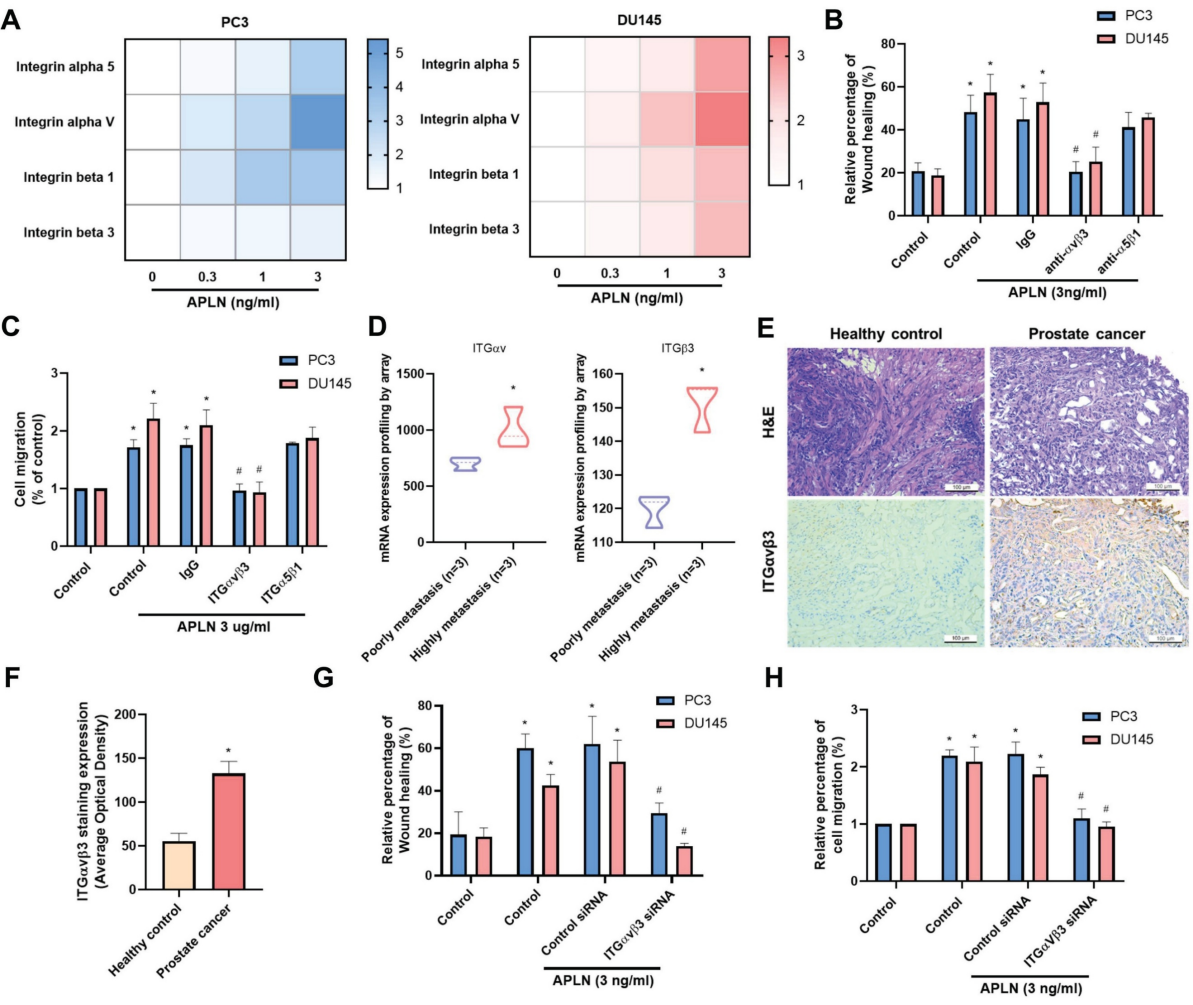
** Apelin enhances integrin αvβ3-dependent prostate cancer motility.** (A) Cells were treated with apelin for 24 hours, the indicated integrin expression was examined by qPCR (n=3). (B&C) Cells were treated with integrin αvβ3 or α5β1 antibody then with apelin, the wound healing and cell migration was examined (n=3). (D) Integrin αv and β3 gene levels in normal and prostate cancer patients retrieved from the GEO database. (E&F) Representative images showing the results of IHC staining for integrin αvβ3 in tissue samples from healthy individuals (n=3) and prostate cancer patients (n=3). (G&H) Cells were co-transfected with integrin αv and β3 siRNA then with apelin, the wound healing and cell migration was examined (n=3). * *p* < 0.05 compared with the control group. # *p* < 0.05 compared with the apelin-treated group.

Apelin has been found to be important for the development of many types of cancer 27, 28, however it is yet unknown how apelin affects prostate cancer metastases. Human prostate cancer tissue samples have considerably higher apelin expression levels than normal tissue samples as determined by H&E and IHC staining (Fig. 1A&B). The data from GEO dataset also confirmed the similar results (Fig. 1C). Next, we used a wound healing migration assay to examine the migratory effects of apelin in prostate cancer cells. Apelin promotes wound healing migration in both PC3 and DU145 prostate cancer cells in a concentration-dependent manner (Fig. 1D-G). However, apelin treatment did not affect the cell viability in prostate cancer cell lines (Supplementary Fig. S1). Integrin level is linked to metastasis and correlates with various stages of human prostate cancer 5. Stimulation of PC3 and DU145 cells enhances the expression of integrins α5, αv, β1, and β3 in a concentration-dependent manner (Fig. 2A). Integrins α5, αv, β1, and β3 form two unique complexes, namely αvβ3 and α5β1. Cells treated with antibodies that block integrin αvβ3, but not α5β1, exhibited inhibited apelin-induced promotion of wound healing and cell migration (Fig. 2B&C). Expression levels of integrins αv and β3 in highly metastatic prostate cancer patient samples from the GEO database were significantly higher than those in patients with poorly metastatic prostate cancer (Fig. 2D). In addition, H&C and IHC staining revealed that integrin αvβ3 levels are higher in human prostate cancer tissue samples than in normal tissue samples (Fig. 2E&F). Co-transfection of cells with integrin αv and β3 siRNAs diminished apelin-promoted cell motility (Fig. 2G&H), indicating that apelin enhances prostate cancer motility by promoting integrin αvβ3 production.

### MAPK and STAT3 signaling pathways are mediated apelin-enhanced integrin expression and cell migration

**Figure 3:**
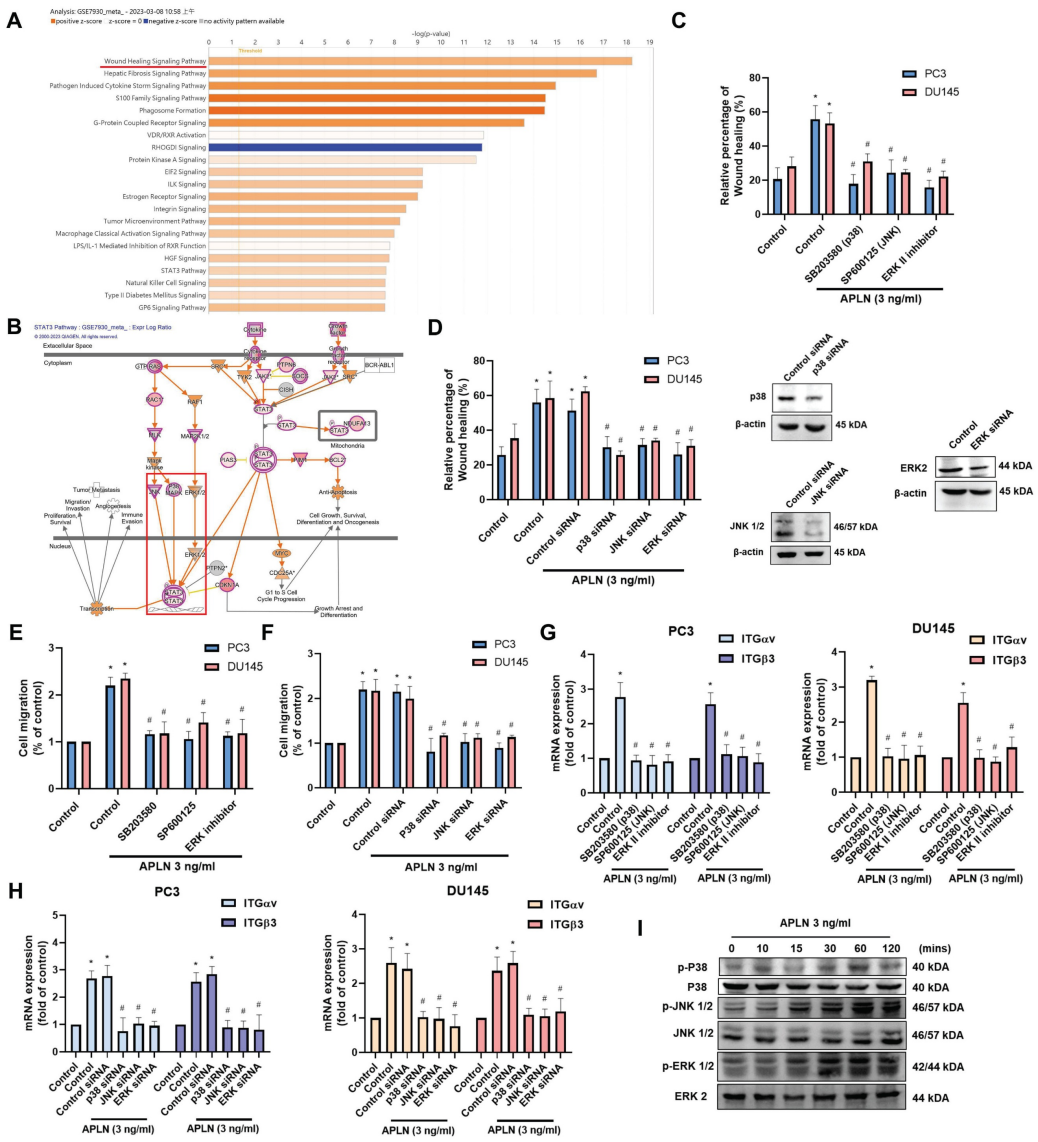
** MAPK pathway is regulated apelin-induced integrin expression and prostate cancer migration.** (A&B) IPA pathway enrichment figure showing pathways that were changed in the GSE7930 dataset (Orange color indicated upregulated gene profile; blue color indicated downregulated gene profile). (C-H) Cells were treated with ERK (ERK II inhibitor; 10 μM), p38 (SB203580; 10 μM) and JNK (SP600125; 10 μM) inhibitors or transfected with ERK, p38 and JNK siRNA for 24 hours then with apelin, the wound healing, cell migration and integrin mRNA expression was examined (n=3). (I) PC3 cells were stimulated with apelin and ERK, p38 and JNK phosphorylation was examined by Western blotting (n=3). * *p* < 0.05 compared with the control group. # *p* < 0.05 compared with the apelin-treated group.

**Figure 4:**
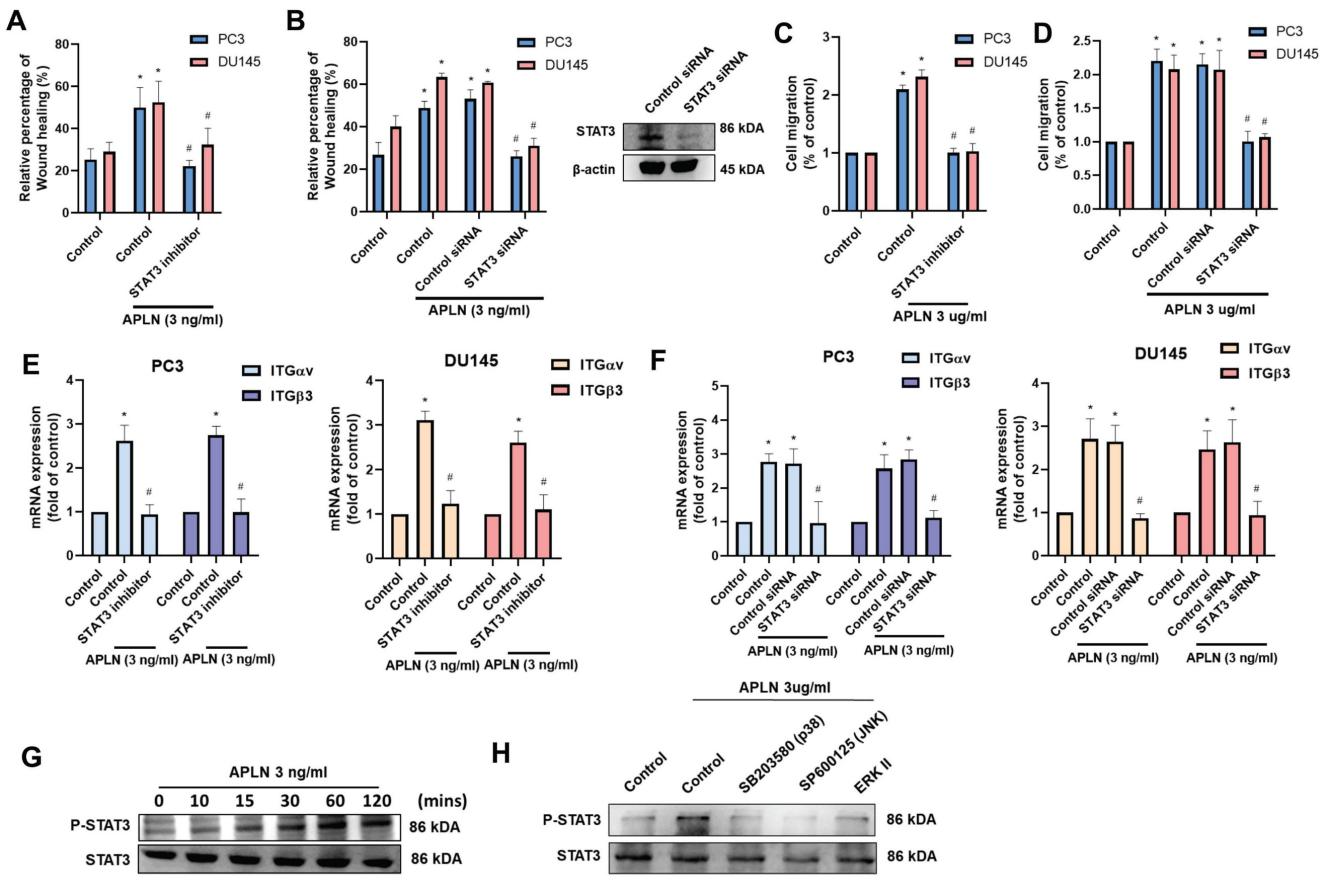
** Apelin promotes integrin production and cell motility via the STAT3 pathway.** (A-F) Cells were treated with STAT3 inhibitor (10 μM) or transfected with STAT3 siRNA for 24 hours then with apelin, the wound healing, cell migration and integrin mRNA expression was examined (n=3). (G&H) PC3 cells were treated with apelin or pretreated with ERK, p38 and JNK inhibitor then with apelin, the STAT3 phosphorylation was examined by Western blotting (n=3). * *p* < 0.05 compared with the control group. # *p* < 0.05 compared with the apelin-treated group.

Next, we use IPA software to examine signaling pathways in the GSE7930 database in order to learn more about the mechanism behind cell motility. The findings showed that the major signaling pathways in the wound healing signaling system are MAPK (ERK, p38, JNK) and STAT3 (Fig. 3A&B). Stimulating cells with ERK (ERK II inhibitor), p38 (SB203580), and JNK (SP600125) inhibitors blocked apelin-induced wound healing and cell migration (Fig. 3C-F). Transfection with ERK, p38, and JNK siRNAs inhibited apelin-promoted cell motility (Fig. 3D&F). These inhibitors and siRNAs also diminished apelin-induced integrin expression (Fig. 3G&H). On the other hand, treatment of PC3 cells with apelin induced phosphorylation of ERK, p38, and JNK (Fig. 3I). Furthermore, both STAT3 inhibitor and STAT3 siRNA reduced apelin-facilitated cell motility and integrin generation (Fig. 4A-F). STAT3 phosphorylation was enhanced in a time-dependent manner after apelin stimulation (Fig. 4G). ERK, p38, and JNK inhibitors reduced apelin-enhanced STAT3 phosphorylation (Fig. 4H). Thus, the MAPK and STAT3 pathways are involved in apelin-induced integrin production and cell motility.

### Apelin enhances integrin-dependent prostate cancer motility by suppressing miR-8070 generation

**Figure 5:**
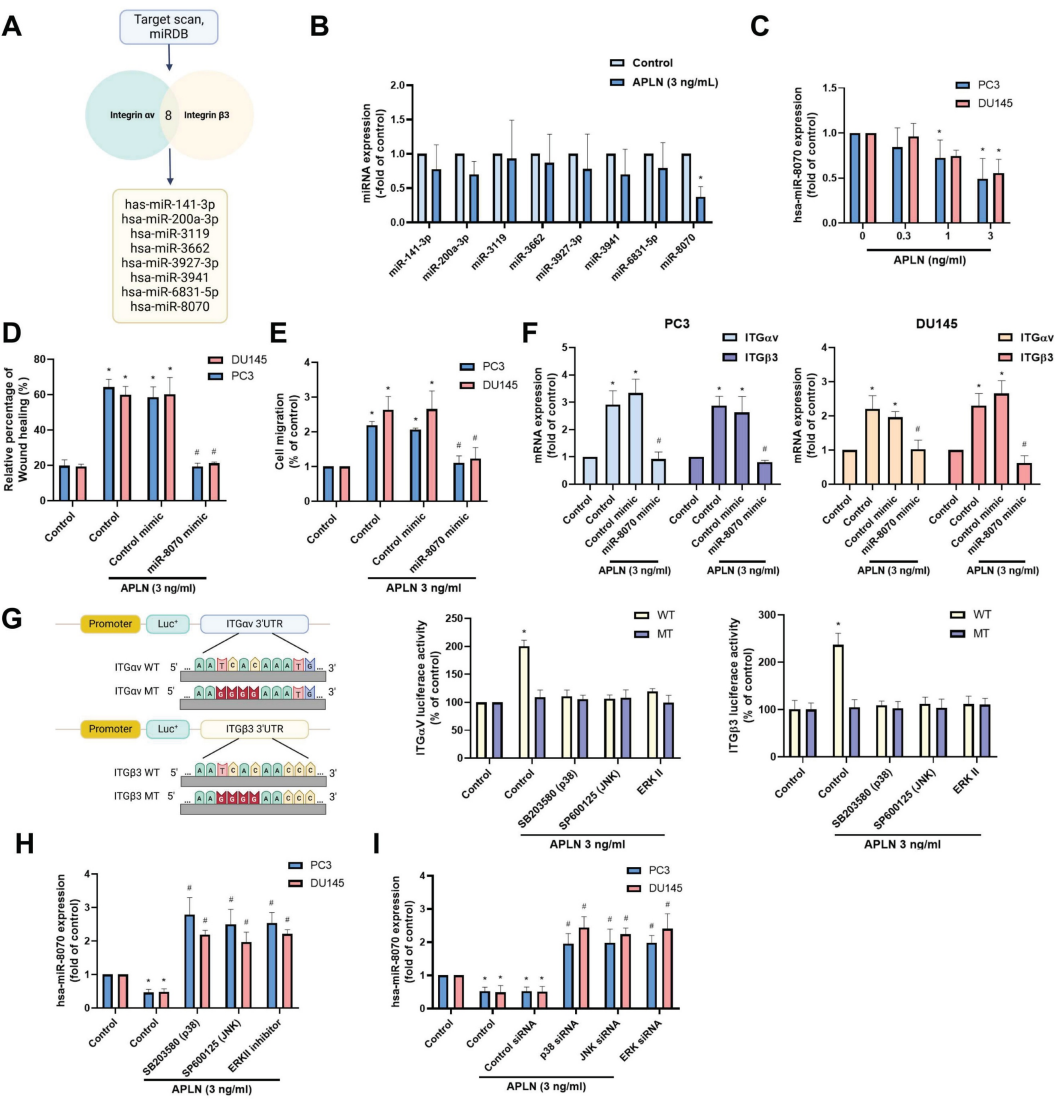
** Apelin enhances integrin expression and promotes cell migration by inhibiting miR-8070 expression.** (A) The diagrams depict the selection of miRNA candidates targeting integrin αv and β3. (B) PC3 cells were treated with apelin, the miRNAs expression was examined by qPCR (n=3). (C) Cells were treated with apelin for 24 hours, the miR-8070 expression was examined by qPCR (n=3). (D-F) Cells were transfected with miR-8070 mimic for 24 hours then with apelin, the wound healing, cell migration and integrin mRNA expression was examined (n=3). (G-I) Cells were treated with ERK, p38 and JNK inhibitor or siRNA then with apelin, the 3'UTR activity and miRNA expression was examined by luciferase activity and qPCR (n=3). * *p* < 0.05 compared with the control group. # *p* < 0.05 compared with the apelin-treated group.

The metastasis of prostate cancer has been linked to dysregulated miRNAs 29. Using two bioinformatics tools (TargetScan and miRDB), we identified eight miRNAs that directly bind to the 3' UTRs of both integrin αv and β3 (Fig. 5A). Among them, apelin treatment predominantly downregulated miR-8070 expression (Fig. 5B). Additionally, incubating PC3 or DU145 cells with apelin led to a concentration-dependent reduction in miR-8070 expression (Fig. 5C). To determine whether apelin enhances integrin expression through the regulation of miR-8070, further experiments were conducted involving the transfection of prostate cancer cells with a miR-8070 mimic, which reversed the effects of apelin on wound healing, cell migration, and integrin synthesis (Fig. 5D-F). Luciferase reporter plasmids containing the wild-type (WT) integrin αv and β3 3'-UTRs, as well as regions with mismatches (MT) predicted for miR-8070, were used to evaluate the influence of miR-8070 on integrin αv and β3 gene transcription. Apelin induced luciferase activity in WT-integrin αv and β3 3'-UTR plasmids but not in MT-integrin αv and β3 3'-UTR plasmids (Fig. 5G). Moreover, incubation with ERK, p38, and JNK inhibitors counteracted the apelin-induced effects on miR-8070 synthesis but not on WT luciferase activity (Fig. 5G-I), suggesting that apelin promotes integrin-dependent prostate cancer motility by suppressing miR-8070 via the MAPK pathway.

### Blocking apelin inhibits metastasis of prostate cancer *in vivo*

**Figure 6:**
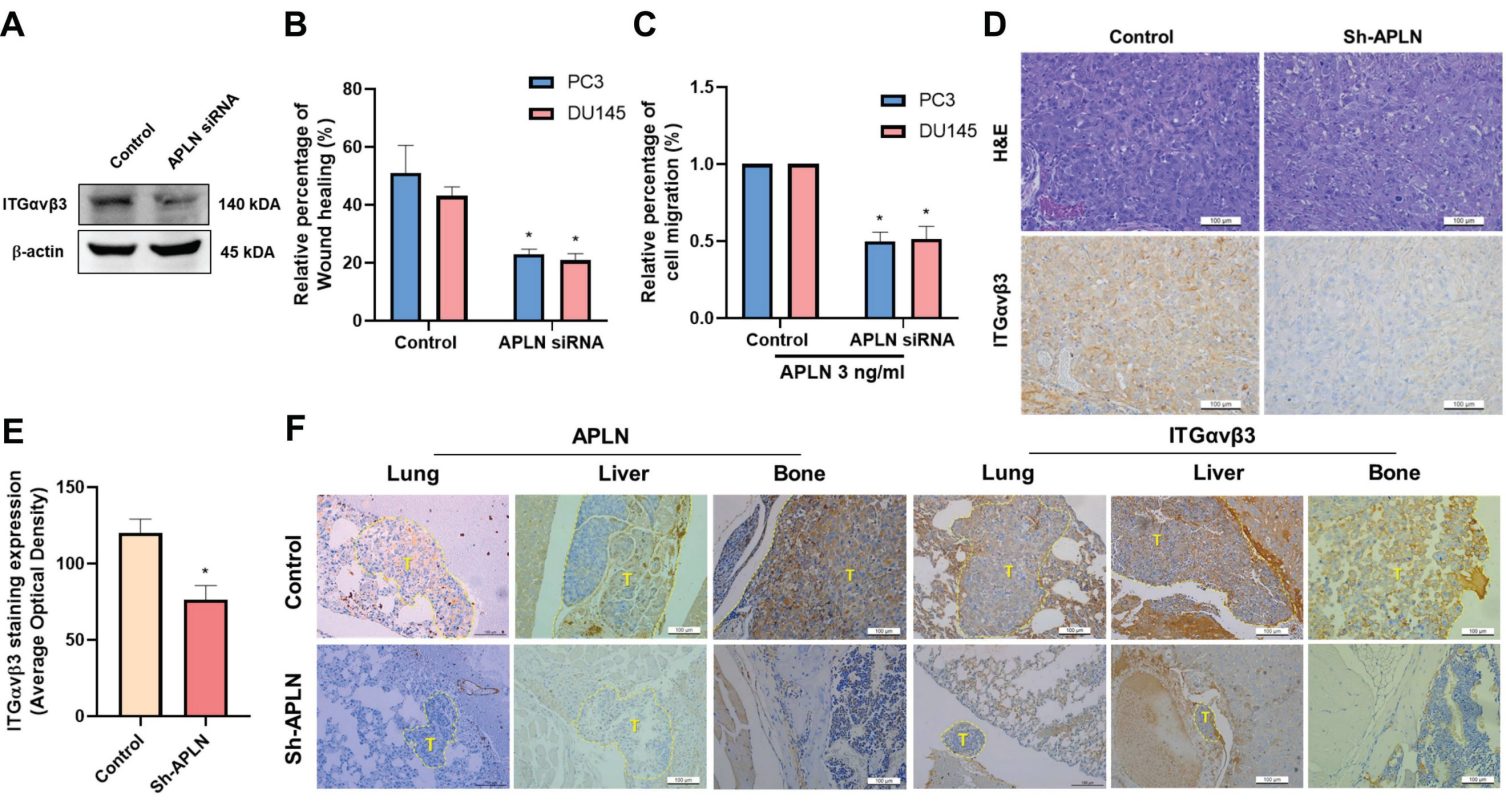
** Apelin blockade inhibits prostate cancer metastasis *in vivo*.** (A-C) Cells were transfected with apelin siRNA, the integrin expression, wound healing and cell migration was examined (n=3). (D&E) IHC analysis of prostate cancer tissue samples stained with integrin αvβ3 antibody (n=3). (F) IHC analysis of leg bone (n=3), liver (n=3), and lung (n=3) sections stained with apelin and integrin αvβ3 antibodies. * *p* < 0.05 compared with the control group.

To confirm the effects of apelin on integrin-dependent cell motility, PC3 cells were transfected with apelin siRNA. This transfection reduced integrin production, wound healing, and cell migration (Fig. 6A-C). We previously reported that apelin blockade reduces prostate cancer metastasis in tumor xenograft mouse models 19, the PC-3 cells were implanted into the anterior prostates of nude mice, and after 7 weeks, apelin blockade suppressed tumor growth by approximately 80% and 34%, as measured by IVIS imaging and manual tumor weight measurements, respectively. *Ex vivo* IVIS analysis revealed that apelin blockade inhibited prostate cancer metastasis to the liver and bone by 50% and 75%, respectively. Additionally, apelin blockade reduced metastatic tumor size in the lung, liver, and bone by 78.9%, 95.6%, and 96.1%, respectively, as determined by H&E staining, suggesting that inhibiting apelin suppresses prostate cancer metastasis. Here, we analyzed samples to examine integrin αvβ3 expression. IHC staining revealed that apelin blockade significantly reduced integrin αvβ3 expression levels in the prostate (Fig. 6D&E). Moreover, apelin blockade inhibited the occurrence of distant metastases and reduced the expression of apelin and integrin αvβ3 in the bone, liver, and lung (Fig. 6F), indicating that the apelin/integrin axis plays a crucial role in prostate cancer metastasis.

## Discussion

Prostate cancer development is significantly influenced by adipose tissue 30. The development, invasion, and metastasis of tumor cells have been thought to be influenced by direct or indirect interactions with adipocytes. In prostate cancer, bone tissue is the most common location of metastasis 31, and several investigations have suggested that bone marrow adipocytes have a role in the development and exacerbation of bone metastases 31. Adipocytes produce the polypeptide apelin, which has a variety of biological functions and aids in tumor spreading by encouraging cell proliferation, motility, and survival 32. It has been demonstrated that ovarian cancer tumor development and metastasis are facilitated by elevated apelin expression in ovarian tumor cells 33. In this study, elevated apelin and integrin αvβ3 expression levels were detected in prostate cancer samples compared to those in normal individuals. Cell culture experiments demonstrated that apelin promotes integrin αvβ3-dependent prostate cancer migration by activating STAT3 and inhibiting miR-8070 via the MAPK pathway. Importantly, the *in vivo* study confirmed that inhibiting apelin reduces integrin expression and prostate cancer metastasis. We propose that the apelin/integrin axis represents a novel therapeutic target for treating metastatic prostate cancer. Our results are based on *in vitro* experiments and preclinical animal models; however, a limitation of this study is that the findings cannot be directly applied to clinical patients. Further development of therapies targeting the apelin/integrin axis will benefit prostate cancer patients undergoing treatment. Apelin is also a critical factor in obesity- and aging-related disorders 32. Whether targeting apelin can also mitigate obesity- and aging-related disorders requires further investigation.

As tumors grow and spread, changes in integrin expression are controlled 10. Prostate cancer cell motility and proliferation in bone are facilitated by increases in integrin expression, indicating that integrin plays a critical role in prostate cancer metastasis to bone 34. Furthermore, integrin makes prostate cancer malignancies more aggressive, and other studies have shown that prostate cancer has high levels of integrin expression, which can activate and cause metastases 34, 35. Therefore, integrin seems to be a suitable target for therapeutic inhibition of metastases of prostate cancer. Our results reveal that apelin increases the production of integrins α5, αv, β1, and β3. An antibody against αvβ3, but not α5β1, blocks apelin-induced cell motility. Genetic inhibition of integrin αvβ3 produces similar effects. Apelin blockade inhibits integrin αvβ3 expression, cell migration, and metastasis both *in vitro* and *in vivo*. Therefore, integrin αvβ3 is a critical mediator of apelin-regulated prostate cancer metastasis.

MAPK signaling pathways are essential for many biological functions, including proliferation, growth, differentiation, and apoptosis 36, 37. According to our IPA analysis of the GSE7930 database, the major wound healing signaling system was linked to the MAPK and STAT3 signaling pathways. We documented that apelin administration enhances the phosphorylation of MAPK molecules, including ERK, p38, and JNK. Treatment with pharmacological inhibitors of ERK, p38, and JNK suppressed apelin-induced integrin expression and cell migration. Transfection with their respective siRNAs produced consistent results. STAT3 transactivation is a key process in regulating tumor metastasis 38, 39. Similarly, both STAT3 inhibitors and siRNA reversed apelin-mediated effects. Apelin-induced STAT3 activation was blocked by treatment with ERK, p38, and JNK inhibitors, indicating that apelin enhances integrin-dependent prostate cancer migration via the MAPK and STAT3 pathways.

At the post-transcriptional level, small, non-coding miRNAs are essential for regulating gene expression l 40. This regulation controls physiological and pathological processes, including cancer, by destroying or inhibiting the translation of target mRNAs 41-43. A promising treatment strategy to stop tumor metastasis is to alter miRNA expression through pharmacological interventions, which may be utilized to stop cancer cells from migrating 44, 45. This study's investigation of miRNA database software revealed that miR-8070 disrupts integrin αv and β3 transcription. Subsequent studies showed that apelin administration decreased miR-8070 expression and that the introduction of a miR-8070 mimic into prostate cancer cells reversed apelin-induced integrin αv and β3 production, thereby reducing cell motility. Additionally, apelin-induced inhibition of miR-8070 synthesis was reversed by blocking ERK, p38, and JNK expression, indicating that apelin regulates integrin production and cell motility in prostate cancer by reducing miR-8070 levels through the MAPK pathway. We demonstrated that the activation of STAT3 and inhibition of miR-8070 via the ERK, p38, and JNK pathways mediate apelin-facilitated integrin synthesis and cell motility. The miRWalk database revealed that STAT3 does not contain an miR-8070 binding site, suggesting that miR-8070 cannot directly regulate STAT3 expression. Whether miR-8070 and STAT3 interact through other mechanisms requires further investigation.

**Figure 7:**
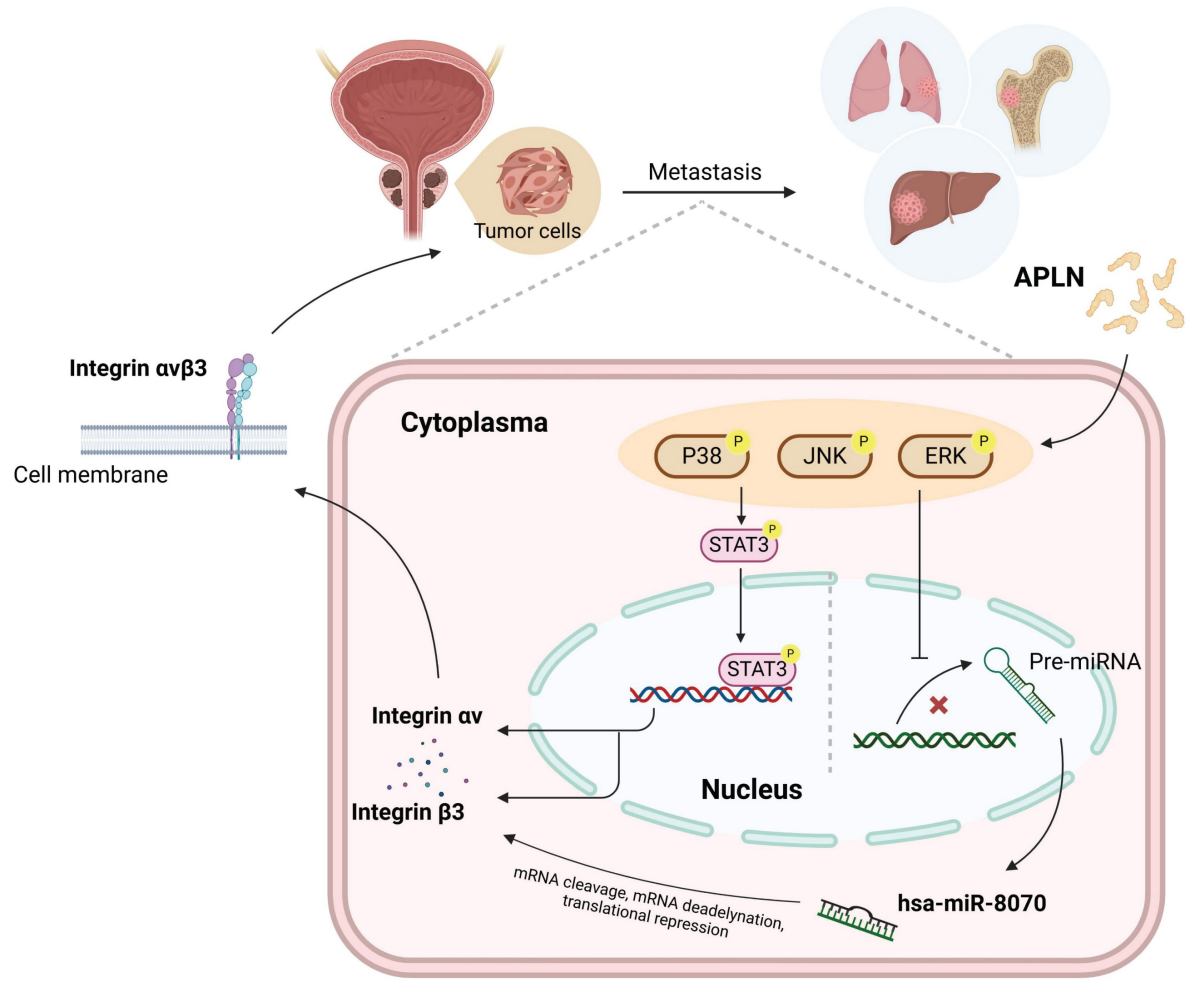
** Schematic diagram illustrating the mechanism underlying the effects of apelin in prostate cancer metastasis.** Apelin stimulation enhances integrin αvβ3-dependent prostate cancer migration and metastasis. The activation of STAT3 and inhibition of miR-8070 via the ERK, p38 and JNK pathways mediate apelin-facilitated integrin synthesis and cell motility.

In conclusion, we reveal that apelin increases integrin αvβ3 synthesis and promotes prostate cancer metastasis by activating STAT3 and inhibiting miR-8070 via the MAPK pathway (Fig. 7). Targeting the apelin/integrin αvβ3 axis may serve as a potential therapeutic strategy for treating metastatic prostate cancer.

## Supplementary Material

Supplementary figure and table.
