# Supplementary material for: Apelin facilitates integrin αvβ3 production and enhances metastasis in prostate cancer by activating STAT3 and inhibiting miR-8070
Source: Int J Biol Sci. 2025 Jun 12;21(9):4117–28. doi: 10.7150/ijbs.113161 (PMC12223769; doi:10.7150/ijbs.113161)
Supplement: Supplementary file 1 — Supplementary figure and table. [file ijbsv21p4117s1.pdf]

Supplementary data

Primer		Squence (5' to 3' direction)
Integrin αv	Forward:	GCTGTCGGAGATTTCAATGGT
	Reverse:	TCTGCTCGCCAGTAAAATTGT
Integrin β3	Forward:	AGTAACCTGCGGATTGGCTTC
	Reverse:	GTCACCTGGTCAGTTAGCGT
GAPDH	Forward:	ACCACAGTCCATGCCATCAC
	Reverse:	TCCACCACCCTGTTGCTGTA

Table S1. The primer sequences used for qPCR.

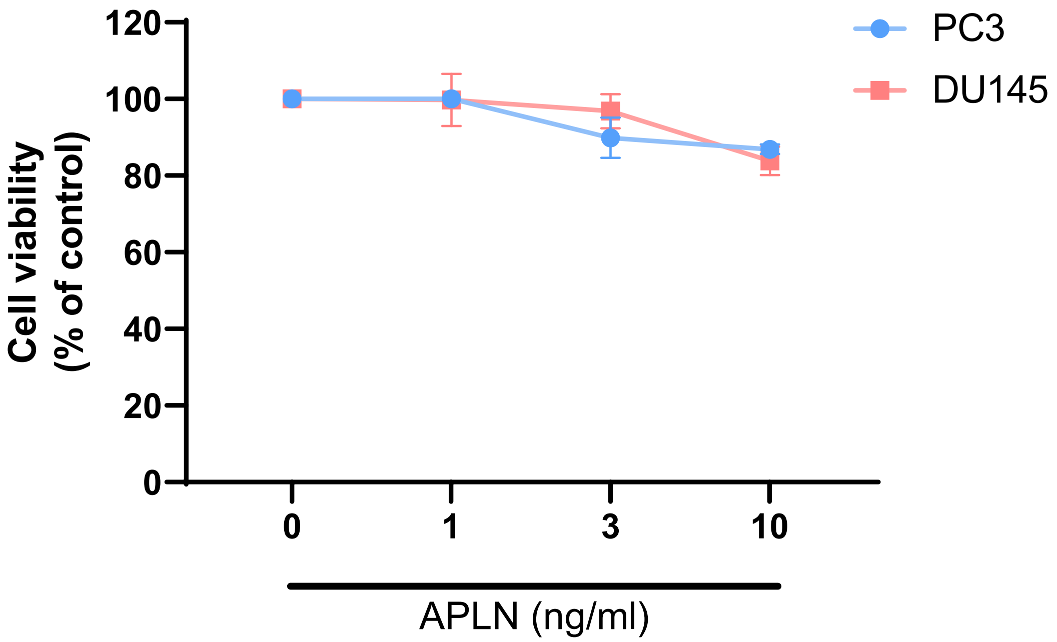

Figure S1. Apelin did not affect cell viability in prostate cancer cells. Cells were treated with apelin for 24 h, the cell viability was examined by MTT assay.
